# Supplementary material for: Modification and analysis of context-specific genome-scale metabolic models: methane-utilizing microbial chassis as a case study
Source: mSystems. 2024 Dec 19;10(1):e01105-24. doi: 10.1128/msystems.01105-24 (PMC11748545; doi:10.1128/msystems.01105-24)
Supplement: Supplemental Figures — Fig. 1.1-1.8. [file msystems.01105-24-s0001.docx]

Supplementary Material

# Supplementary Figures


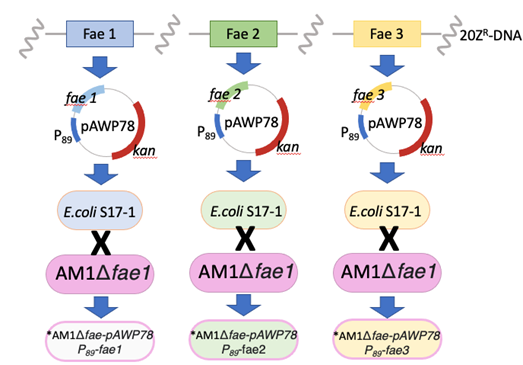


**Supplementary Figure 1.1.** Schematic representation of plasmid construction and integration of *fae1* and *fae* homologues from *M. alcaliphilum* 20Z^R^ into *M. extorquens* AM1Δ*fae1* mutant.


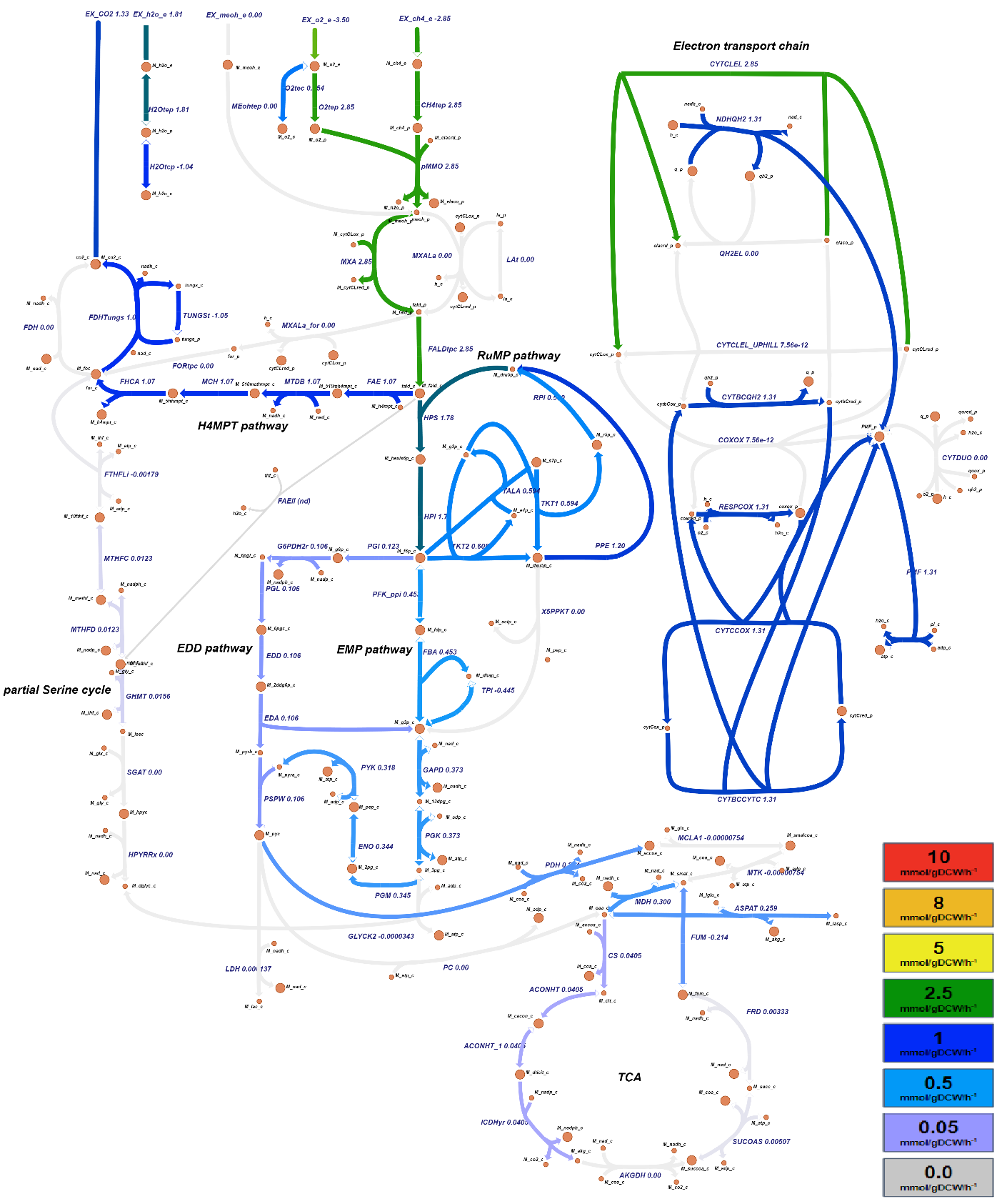


**Supplementary** **Figure 1.2.** The fluxes distribution predicted by original *i*IA409 model for growth on CH_4_ in the presence of Ca, W, Cu. The line thickness and color indicate the extent of the flux rate through the reaction in mmol*gDCW^-1^*h^-1^.


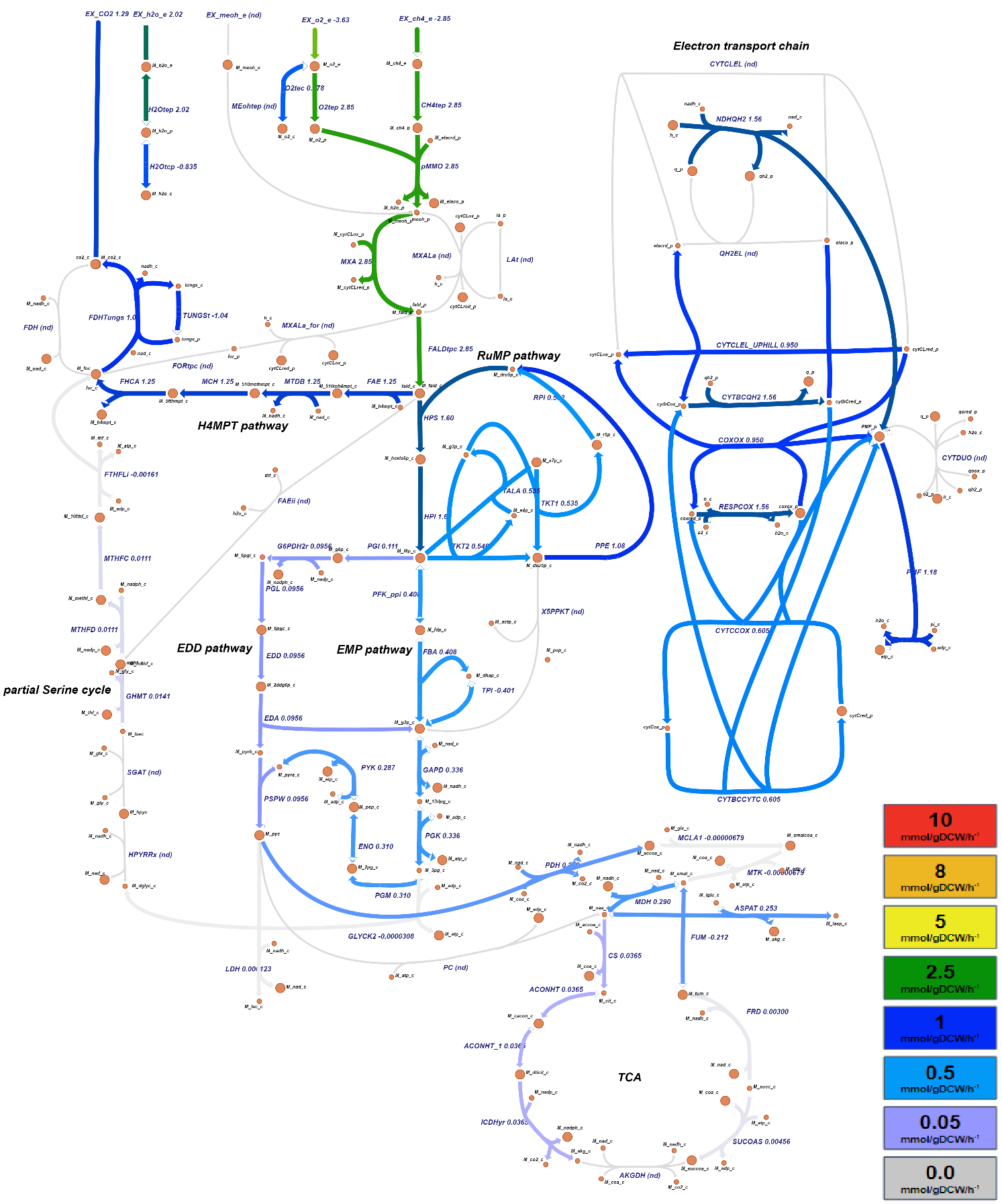


**Supplementary Figure 1.3.** The fluxes distribution predicted by CS model based on transcriptomic counts data for growth on CH_4_ in the presence of Ca, W, Cu. The line color indicates the extent of the flux rate through the reaction in mmol*gDCW^-1^*h^-1^.


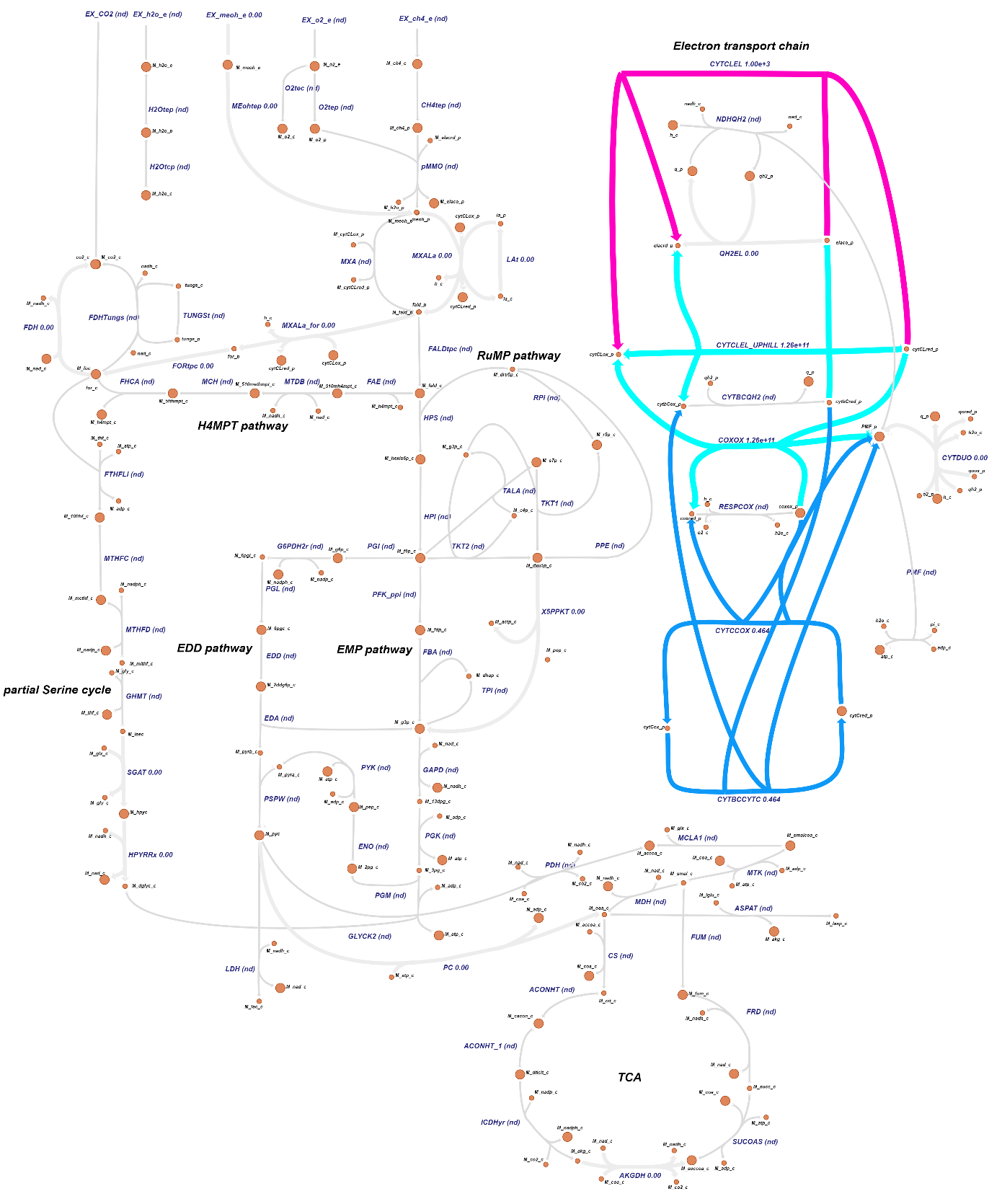


**Supplementary Figure 1.4.** DRFs between *i*IA409 and CS model based on transcriptomic counts data for growth on CH_4_ in the presence of Ca, W, Cu. The line color indicates the ratio of the reaction flux in the CS model to reaction flux in *i*IA409. Reactions are assigned a value of 1000 if the reaction is turned off in the CS model, while it is active in the original one, and 2000 if it is conversely.


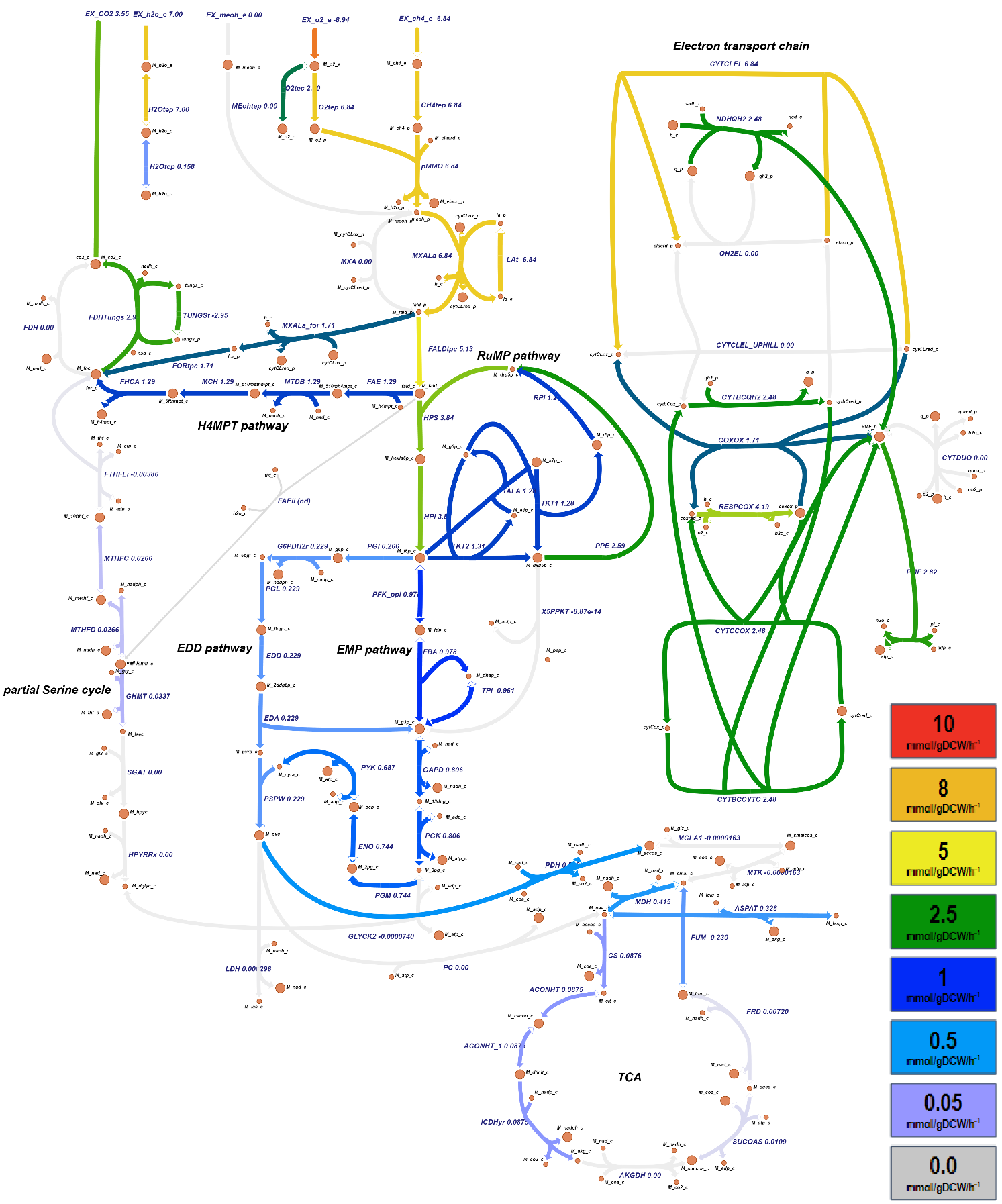


**Supplementary Figure 1.5.** The fluxes distribution predicted by original *i*IA409 model for growth on CH_4_ in the presence of La, W, Cu. The line color indicates the extent of the flux rate through the reaction in mmol*gDCW^-1^*hr^-1^.


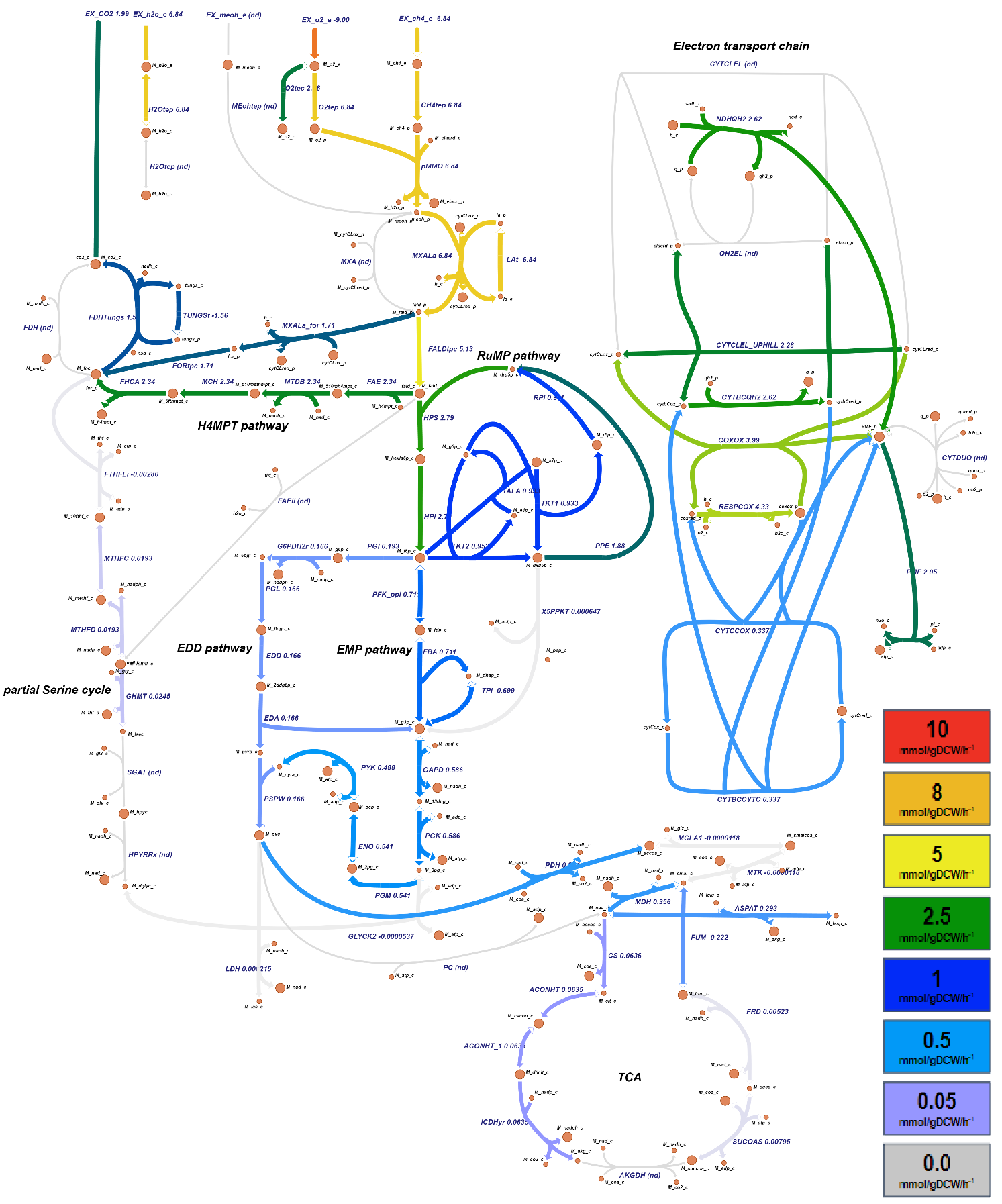


**Supplementary Figure 1.6.** The fluxes distribution predicted by CS-model based on transcriptomic counts data for growth on CH_4_ in the presence of La, W, Cu. The line color indicates the extent of the flux rate through the reaction in mmol*gDCW^-1^*hr^-1^.


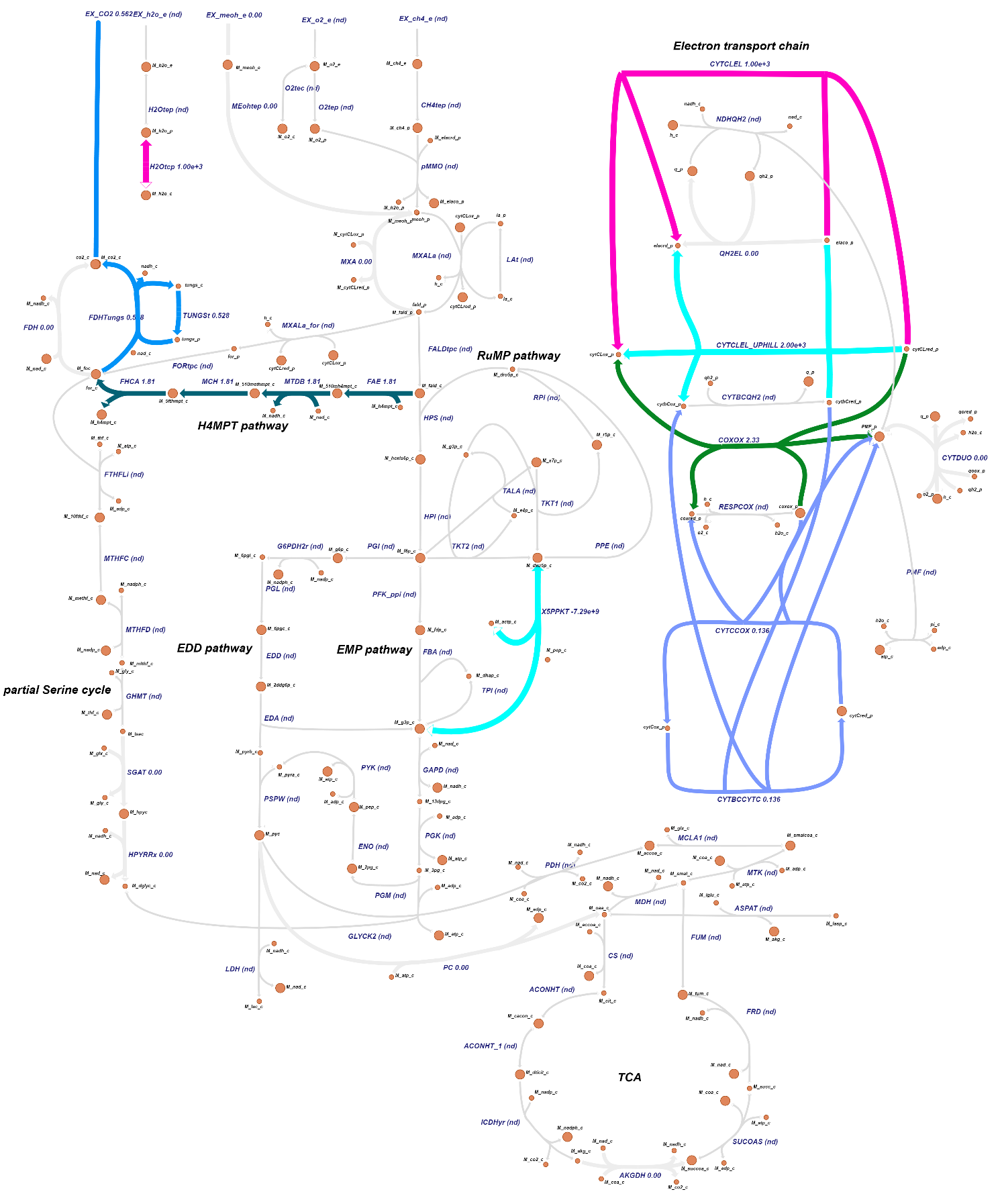


**Supplementary Figure 1.7.** DRFs between *i*IA409 and CS model based on transcriptomic counts data for growth on CH_4_ in the presence of La, W, Cu. The line color indicates the ratio of the reaction flux in the CS model to reaction flux in *i*IA409. Reactions are assigned a value of 1000 if the reaction is turned off in the CS model, while it is active in the original one, and 2000 if it is conversely.


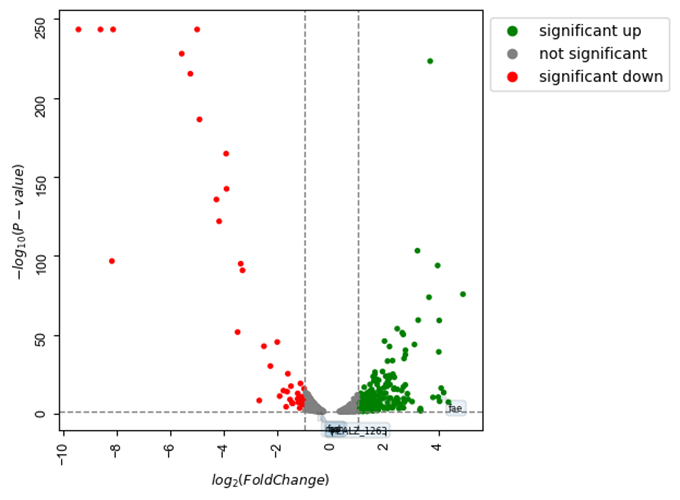


**Supplementary Figure 1.8.** Volcano plot of DEGs between growth on CH_4_ in the presence of La, W, Cu vs growth on CH_4_ in the presence of Ca, W, Cu. Genes names on the plot associated with reactions in H_4_MPT pathway are indicated. Down-expressed genes are marked by red color, while up-expressed genes are indicated by green color (|log2Fold| > 1.5, p_adj_ <0.05).
